# Identifying common trends and ecosystem states to inform Gulf of Alaska ecosystem-based fisheries management

**DOI:** 10.1371/journal.pone.0324154

**Published:** 2025-06-06

**Authors:** Bridget E. Ferriss, Mary E. Hunsicker, Eric J. Ward, Michael A. Litzow, Lauren Rogers, Matt Callahan, Wei Cheng, Seth L. Danielson, Brie Drummond, Emily Fergusson, Christine Gabriele, Kyle Hebert, Russell R. Hopcroft, Jens Nielsen, Kally Spalinger, William T. Stockhausen, Wesley W. Strasburger, Shannon Whelan

**Affiliations:** 1 Resource Ecology and Fisheries Management Division, Alaska Fisheries Science Center, National Marine Fisheries Service, National Oceanic and Atmospheric Administration, Seattle, Washington, United States of America; 2 Fish Ecology Division, Northwest Fisheries Science Center, National Marine Fisheries Service, National Oceanic and Atmospheric Administration, Newport, Oregon, United States of America; 3 Conservation Biology Division, Northwest Fisheries Science Center, National Marine Fisheries Service, National Oceanic and Atmospheric Administration, Seattle, Washington, United States of America; 4 Shellfish Assessment Program, Resource Assessment and Conservation Engineering Division, Alaska Fisheries Science Center, National Marine Fisheries Service, National Oceanic and Atmospheric Administration, Kodiak, Alaska, United States of America; 5 Resource Assessment and Conservation Engineering Division, Alaska Fisheries Science Center, National Marine Fisheries Service, National Oceanic and Atmospheric Administration, Seattle, Washington, United States of America; 6 Pacific States Marine Fisheries Commission, Alaska Fish Information Network, Juneau, Alaska, United States of America; 7 Pacific Marine Environmental Laboratory, National Oceanic and Atmospheric Administration, Seattle, Washington, United States of America; 8 College of Fisheries and Ocean Sciences, University of Alaska Fairbanks, Fairbanks, Alaska, United States of America; 9 United States of America Fish and Wildlife Service, Alaska Maritime National Wildlife Refuge, Homer, Alaska, United States of America; 10 Auke Bay Laboratories Division, Alaska Fisheries Science Center, National Marine Fisheries Service, National Oceanic and Atmospheric Administration, Juneau, Alaska, United States of America; 11 Glacier Bay National Park and Preserve, Gustavus, Alaska, United States of America; 12 Alaska Department of Fish and Game, Commercial Fisheries Division, Juneau, Alaska, United States of America; 13 Cooperative Institute for Climate, Ocean, and Ecosystem Studies, University of Washington, Seattle, Washington, United States of America; 14 Alaska Department of Fish and Game, Commercial Fisheries Division, Kodiak, Alaska, United States of America; 15 Institute for Seabird Research and Conservation, Anchorage, Alaska, United States of America; University of Connecticut, UNITED STATES OF AMERICA

## Abstract

Ecosystem-based fisheries management requires the successful integration of ecosystem information into the fisheries management process. In the Northeast Pacific Ocean, ecosystem data collection and accessibility have achieved successful milestones, yet application to the harvest specification process remains challenging. The synthesis, interpretation, and application of ecosystem information to groundfish fisheries management in the Gulf of Alaska (GOA) can be supported by the identification of common ecosystem trends and ecosystem states across a diverse set of indicators. In this study, we used Dynamic Factor Analysis (DFA) and hidden Markov models (HMM) to analyze 92 indicators in climate, lower-trophic, mid-trophic, and seabird models for the western and eastern GOA marine ecosystems. Time series ranged from 25 to 52 years in length, analyzed through 2022. The DFA identified common trends across indicators and groups of covarying indicators (e.g., biomass of zooplankton species), highlighting opportunities to streamline communication of these data to management. Non-stationarity analyses revealed past changes in relationships, and can provide early warnings in future annual updates if previously identified correlations change. The HMM identified two to three ecosystem states in each sub-model that largely aligned with previously observed long- and short-term shifts in ecosystem dynamics in the region (i.e., shifts starting in 1975, 1988, and 2014). Annually updating these analyses, within an existing framework of reporting ecosystem information to management bodies, can streamline communication and improve early warning of changes in ecosystem dynamics. These tools can provide ecosystem support to management decisions relative to groundfish productivity and resulting harvest specifications.

## Introduction

The successful application of ecosystem-based fisheries management includes the timely acquisition of ecosystem information and the incorporation of that information into the fisheries management process [1]. Data on physical ocean conditions, primary and secondary production and the availability of prey, predators, and competitors can give critical insight into climate and ecosystem processes, and help identify current or emerging impacts on fishery productivity. A common challenge in data-rich systems is the ability to clearly synthesize and operationalize this ecosystem information to best inform harvest management decisions [2]. While some environmental information is included directly into stock assessment models, the majority of ecosystem information is integrated into the harvest specification process through adjacent products with varying levels of success [1,3,4]. Ecosystem information external to the stock assessment models has the advantage of capturing near real time ecosystem changes (versus time lagged recruitment responses in stock assessment models) and can be used to communicate ecosystem status and trends in rapidly changing and non-stationary environments [1]. However, resource managers are constrained in their application of ecosystem information by limited time available to rapidly digest a broad suite of indicators, and limited ability to connect ecosystem dynamics directly to harvest specification decisions [5]. There is a need for tools that can rapidly synthesize a wide breadth of ecosystem information in a way that can be directly tied to fisheries management decisions [6,7].

Non-stationary change presents a challenge for scientists and decision makers [8]. While there are many types of non-stationary change, we differentiate two forms of temporal change. In the first, non-stationary change may result in a change in the mean or variance of a variable of interest; this may include a trend in population abundance or demographic rates, or shift in environmental variables through time. For multiple time series, non-stationary dynamics may result in a shift in the ecosystem state [9]. Our second form of non-stationarity is changing relationships through time; examples include changing predator-prey relationships, or changing relationships between physical environmental drivers and biological responses. While marine ecosystems around the world have experienced both types of non-stationary change, higher latitude regions are experiencing greater rates of change than equatorial regions [10].

The Gulf of Alaska (GOA), in the Northeast Pacific Ocean, has benefitted from a relatively rich network of marine ecosystem data collection and sharing, in support of groundfish fisheries management. The GOA has experienced documented ecosystem state shifts (1976/1977) and other extreme environmental events, such as the multi-year marine heatwave starting in 2014, that have influenced populations of commercially valuable groundfish [11,12]. These extreme environmental events and fishery responses have promoted the continued development and integration of ecosystem data into the annual fishery harvest specification process through stock assessment models, stock-specific Ecosystem and Socio-economic Profiles [13], stock-specific risk tables [14], and large marine ecosystem-specific Ecosystem Status Reports [15]. Scientists and fisheries managers continue to strive to identify and improve the most effective synthesis and application of the information in these products to support management decisions under rapidly changing ocean conditions and fish stock dynamics.

Integrating ecosystem information into fishery management requires a prioritization and selection of appropriate data relative to a given fish species or ecosystem question [16–18]. A broad, and sometimes redundant, suite of ecosystem information is often reported to account for incomplete temporal or spatial monitoring, poorly understood mechanistic relationships, and ease of data availability. Identifying common trends in ecosystem variability, and resulting ecosystem states, can provide a tool for resource managers to assess a breadth of ecosystem information through an applied fisheries management lens. Dynamic Factor Analysis (DFA) is a statistical tool that identifies suites of commonly varying indicators and latent trends in ecosystem variability [19]. Latent trends are unobserved patterns of ecosystem change that can be identified through statistical models of observed data. DFA can assist in reducing redundancy and simplifying the communication of ecosystem status and trends, supporting greater understanding and facilitating incorporation into management decisions [9,20].

Integration and regular updating of multiple ecosystem time series can also be used to identify ecosystem states and temporal shifts among these states. While there have been advances in the development and selection of ecosystem indicators, their application to trends and status of fish stocks and their ecosystems is still developing [21]. Early warnings or short-term forecasts of shifts in ecosystem state can support annual fishery harvest decisions through stock-specific dynamic reference points [22], ecosystem-level decisions [23], and the identification of thresholds and tipping points [24]. An ecosystem shift is difficult to detect in real time, without tools that can be quickly updated, and that can differentiate background variability across multiple ecosystem components from systemic changes that qualify as a shift to a new state. Hidden Markov Models (HMM) can add value by identifying potential ecosystem states within DFA trends, helping identify the relative magnitude of change in ecosystem trends and allowing comparability to past states [9,20].

The purpose of this study was to assess how data synthesis with appropriate time series tools can be incorporated into an ecosystem management framework, to more effectively synthesize and interpret existing ecosystem indicators used in Gulf of Alaska fisheries management. Specifically, our objectives were to (1) develop ecosystem-level indicators and (2) identify and detect changes in ecosystem states. Distilling these ecosystem indicators to reduce redundancy, identify ecosystem states, and clarify communication of ecosystem information at a spatial and temporal scale relevant to fisheries management decisions is key to advancing ecosystem-based fisheries management in the GOA.

## Methods

### Data and workflow

**Fig 1:**
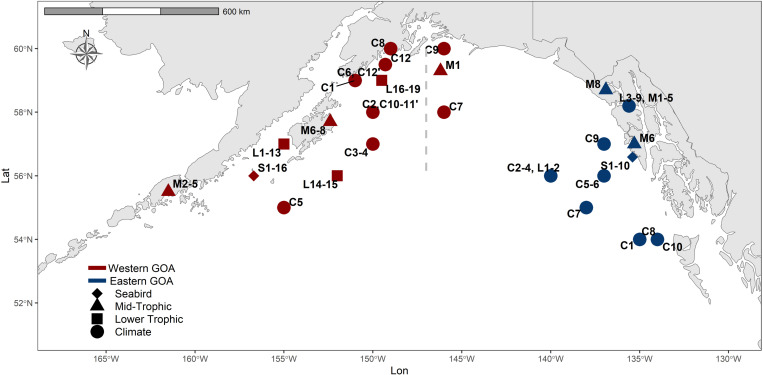
A map of the Gulf of Alaska (GOA), identifying locations of each indicator included in the climate (circles), lower trophic (squares), mid-trophic (triangles), seabird (diamond) and all biology DFA models and HMM analyses. The indicators were divided into western GOA (red) and eastern GOA (blue) models. The label locations have been slightly shifted to avoid overlap in the figure. Labels are defined in Table 1.

Indicator time series were selected based on their inclusion in the GOA Ecosystem Status Report [15], their sensitivity to short-term ecosystem dynamics, the time series length, a preference for empirical versus model-based data, and limited redundancy with other time series (Tables 1 and 2). Indicators were divided into western (approximately 147⁰W to Unimak Pass) and eastern GOA (147⁰W to the Alaska-Canada border) reflecting documented oceanographic and ecological differences between the two sub-regions (Fig 1) [25]. To summarize broad patterns of non-stationary change, indicators were analyzed in six models including climate (e.g., ocean temperature), lower trophic (e.g., biomass of zooplankton and larval fish), mid-trophic (e.g., forage fish abundance), seabird (e.g., reproductive success), and all-biology (subset of species from each biological model) in each region. Examination of trends in a particular collection of time series (e.g., lower trophic indicators) can be used to evaluate non-stationary mean values, but cannot be used to quantify non-stationary relationships between indicators. A novel contribution of our analysis is a comparison of model results across time windows of different lengths which is used to evaluate observational support for time-varying relationships. We can then examine whether the correlation between individual time series and ecosystem trends is changing through time.

**Table 1:** Description of time series included in the climate, lower trophic, mid-trophic, and seabird models in the western GOA. Names with an asterisk were included in the all biology model. The geographic locations of the time series are shown in Fig 1.

Map Ref.	Name	Units	Source	Notes
Climate Model				
C1	Direction of spring Shelikof winds	Degrees	National Buoy Data Center	Mean April-May wind direction at AMAA2 mooring. Units are degrees (out of 365; North = 0 increasing clockwise)
C2	Surface temperature shelf waters (sat.)	^o^C	NOAA Coral Reef Watch Program	Satellite-derived data (Coral Reef Watch Program); mean of daily data averaged (Jun-Aug) over WGOA shelf (surface over 10m-200m depth)
C3	Surface temperature shelf waters (survey)	^o^C	NOAA Bottom Trawl Survey	Survey dates vary within May-Aug; average temperature across shelf (1m-5m)
C4	Shelf ocean temperature at depth (195m-205m)	^o^C	NOAA Bottom Trawl Survey	Survey dates vary within May-Aug; average across shelf
C5	Shelf edge ocean temperature at depth (246m - 255m)	^o^C	NOAA Longline Survey	Summer temperatures average along shelf edge; upper continental slope
C6	Surface temperatures along Seward Line	^o^C	Seward Line Survey	Mean surface temperatures (0-10m) along Seward Line transect in May (mouth of Resurrection Bay near Seward to the outer continental slope of the northern GOA)
C7	Eddy Kinetic Energy	cm^2^· s^-2^		Mean eddy kinetic energy (Dec, Jan, Feb) calculated from sea level height anomalies (gridded altimetry data); Region C is a local maxima along the shelf off Kodiak Island (altimetry record since 1992)
C8	N60W149 upwelling	m^3^· s^-1^· 100m coastline	Pacific Fisheries Environmental Laboratory	Mean values from June, July, August (season when downwelling relaxes)
C9	N60W146 upwelling	m^3^· s^-1^· 100m coastline	Pacific Fisheries Environmental Laboratory	Mean values from June, July, August (season when downwelling relaxes)
C10	winter surface temperatures	^o^C	ERSST	Mean monthly anomalies (November, December, January, February, March) of western GOA shelf water
C11	spring surface temperatures	^o^C	ERSST	Mean monthly anomalies (April, May, June) of western GOA shelf water
C12	GAK1 spring 20m salinity	ppt	University of Alaska	Mean values for February, March, April, adjusted for mean sampling date
Lower Trophic Model				
L1	*Larval *Ammodytes personatus*	#· 10m^-2^	NOAA ECOFOCI survey	Mean abundance
L2	*Larval *Atheresthes stomias*	#· 10m^-2^	NOAA ECOFOCI survey	Mean abundance
L3	*Larval *Gadus chalcogramma*	#· 10m^-2^	NOAA ECOFOCI survey	Mean abundance
L4	*Larval *Hippoglossoides elassodon*	#· 10m^-2^	NOAA ECOFOCI survey	Mean abundance
L5	*Larval *Lepidopsetta polyxystra*	#· 10m^-2^	NOAA ECOFOCI survey	Mean abundance
L6	*Larval *Sebastes* spp.	#· 10m^-2^	NOAA ECOFOCI survey	Mean abundance
L7	Larval *Bathymaster* spp.	#· 10m^-2^	NOAA ECOFOCI survey	Mean abundance
L8	Larval *Gadus macrocephalus*	#· 10m^-2^	NOAA ECOFOCI survey	Mean abundance
L9	Larval *Hippoglossus stenolepis*	#· 10m^-2^	NOAA ECOFOCI survey	Mean abundance
L10	Larval *Lepidopsetta bilineata*	#· 10m^-2^	NOAA ECOFOCI survey	Mean abundance
L11	Larval *Platichthys stellatus*	#· 10m^-2^	NOAA ECOFOCI survey	Mean abundance
L12	Larval *Sebastes* spp.	#· 10m^-2^	NOAA ECOFOCI survey	Mean abundance
L13	Larval *Stenobrachius leucopsarus*	#· 10m^-2^	NOAA ECOFOCI survey	Mean abundance
L14	*Chl-a concentration	ug· L^-1^		Satellite-derived chla was calculated as an average for April-June, using compiled 8-day satellite chlorophyll-a at a 4 km-resolution from The Hermes GlobColour website (http://hermes.acri.fr/) [26]. This is a standardized merged Chl-a product, combining remote sensing data from SeaWiFS, MERIS, MODIS, VIIRS and OLCI. All calculations were done across the shelf (10m-200m) for the EGOA/WGOA and starting 3 miles offshore
L15	*Chl-a timing of peak bloom	day of year		Satellite-derived chla was calculated as an average for April-June, using compiled 8-day satellite chlorophyll-a at a 4 km-resolution from The Hermes GlobColour website (http://hermes.acri.fr/) [26]. This is a standardized merged Chl-a product, combining remote sensing data from SeaWiFS, MERIS, MODIS, VIIRS and OLCI. These data were also used to estimate the timing (day of year) of the maximum spring peak. All calculations were done across the shelf (10m-200m) for the EGOA/WGOA and starting 3 miles offshore
L16	*Small calanoid copepod spring biomass	g· m^-3^ wet weight	Seward Line Survey	Transects south of Seward during the first 10 days of May. Data are averaged over the top 100 m of the water column to estimate wet-weight biomass of calanoid copepods and euphausiids. Small copepods are from a 0.15mm mesh net. Large copepods are from a 0.5 mm mesh net
L17	*Large calanoid copepod spring biomass	g· m^-3^ wet weight	Seward Line Survey	
L18	Large calanoid copepod fall biomass	g· m^-3^ wet weight	Seward Line Survey	Transects south of Seward. Data are averaged over the top 100 m of the water column to estimate wet-weight biomass of calanoid copepods and euphausiids. Small copepods are from a 0.15mm mesh net. Large copepods are from a 0.5 mm mesh net
L19	Small calanoid copepod fall biomass	g· m^-3^ wet weight	Seward Line Survey	
Mid-Trophic Model				
M1	*Proportion of capelin in rhinoceros auklet chick diets	proportion	Institute for Seabird Research and Conservation	Diet analysis of seabird colonies in summer on Middleton Island
M2	*Pavlof Bay pink shrimp abundance	CPUE	Alaska Department of Fish and Game small mesh trawl	log (x + 0.01) transformed
M3	*Pavlof Bay capelin abundance	CPUE	Alaska Department of Fish and Game small mesh trawl	log (x + 0.01) transformed
M4	Pavlof Bay eulachon abundance	CPUE	Alaska Department of Fish and Game small mesh trawl	log (x + 0.01) transformed
M5	Pavlof Bay jellyfish abundance	CPUE	Alaska Department of Fish and Game small mesh trawl	log (x + 0.01) transformed
M6	*Chiniak Bay pink shrimp abundance	CPUE	Alaska Department of Fish and Game small mesh trawl	log (x + 0.01) transformed
M7	*Chiniak Bay eulachon abundance	CPUE	Alaska Department of Fish and Game small mesh trawl	log (x + 0.01) transformed
M8	*Chiniak Bay jellyfish abundance	CPUE	Alaska Department of Fish and Game small mesh trawl	log (x + 0.01) transformed
Seabird Model				
S1	*Tufted puffin reproductive success	proportion	US Fish and Wildlife Service colony surveys	Diving piscivore seabird; proportion of nests with fledged chicks from eggs laid (Chowiet Island)
S2	*Black-legged kittiwake reproductive success	proportion	US Fish and Wildlife Service colony surveys	Surface piscivore (some plankton); proportion of nests with fledged chicks from the total number of nests built (Chowiet Island)
S3	*Parakeet auklet reproductive success	proportion	US Fish and Wildlife Service colony surveys	Diving planktivore; proportion of nests with fledged chicks from eggs laid (Chowiet Island)
S4	*Parakeet auklet mean hatch date	day of year	US Fish and Wildlife Service colony surveys	Diving planktivore (Chowiet Island). Jan1 = Day1
S5	*Tufted puffin mean hatch date	day of year	US Fish and Wildlife Service colony surveys	Diving piscivore seabird (Chowiet Island). Jan1 = Day1
S7	Common murre reproductive success	proportion	US Fish and Wildlife Service colony surveys	Diving piscivore; proportion of nests with fledged chicks from the from eggs laid (Chowiet Island)
S8	Thick-billed murre reproductive success	proportion	US Fish and Wildlife Service colony surveys	Diving piscivore; proportion of nests with fledged chicks from eggs laid (Chowiet Island)
S9	Horned puffin reproductive success	proportion	US Fish and Wildlife Service colony surveys	Diving piscivore; proportion of nests with fledged chicks from eggs laid (Chowiet Island)
S12	Common murre mean hatch date	day of year	US Fish and Wildlife Service colony surveys	Diving piscivore seabird (Chowiet Island). Jan1 = Day1
S13	Thick-billed murre mean hatch date	day of year	US Fish and Wildlife Service colony surveys	Diving piscivore seabird (Chowiet Island). Jan1 = Day1
S14	Horned puffin mean hatch date	day of year	US Fish and Wildlife Service colony surveys	Diving piscivore seabird (Chowiet Island). Jan1 = Day1
S15	Glaucous-winged gull mean hatch date	day of year	US Fish and Wildlife Service colony surveys	Surface piscivore seabird (Chowiet Island). Jan1 = Day1
S16	*Black-legged kittiwake mean hatch date	day of year	US Fish and Wildlife Service colony surveys	Surface piscivore (some plankton). (Chowiet Island). Jan1 = Day1

**Table 2:** Description of time series included in the climate, lower trophic, mid-trophic, and seabird models in the eastern Gulf of Alaska. Names with an asterisk were included in the all biology model. The geographic locations of the time series are specified in Fig 1.

Map Ref.	Name	Units	Source	Notes
Climate Model				
C1	Papa Trajectory Index	^o^N	OSCURS model	The latitude of the trajectory endpoint, derived from an OSCURS model (Ocean Surface CURrent Simulator); simulated surface drifter released from Station Papa and run for 90 days from Dec. 1 (based on speed and direction of water movement)
C2	Summer surface temperatures (sat.)	^o^C	NOAA Coral Reef Watch Program	Satellite-derived data (Coral Reef Watch Program); mean of daily data averaged (Jun-Aug) over WGOA shelf (surface over 10m-200m depth)
C3	Winter surface temperatures (sat.)	^o^C	ERSST	Mean monthly temperature anomalies (November, December, January, February, March)
C4	Spring surface temperatures (sat.)	^o^C	ERSST	Mean monthly temperature anomalies (April, May, June)
C5	Shelf surface temperature (1m-5m)	^o^C	NOAA Bottom Trawl Survey	Survey dates vary within May-Aug; average across shelf
C6	Shelf ocean temperature (195m-205m)	^o^C	NOAA Bottom Trawl Survey	Survey dates vary within May-Aug; average across shelf
C7	Shelf edge ocean temperature (246m - 255m)	^o^C	NOAA Longline Survey	Summer temperatures average along shelf edge; upper continental slope
C8	Eddy Kinetic Energy	cm^2^· s^-2^		Mean eddy kinetic energy (Dec, Jan, Feb) calculated from sea level height anomalies (gridded altimetry data); Region A is the ‘Haida Eddy’ (altimetry record since 1992)
C9	Summer upwelling (N57W137)	m^3^· s^-1^ 100m coastline	Pacific Fisheries Environmental Laboratory	Mean values from June, July, August (season when downwelling relaxes)
C10	Summer upwelling (N54W134)	m^3^· s^-1^ 100m coastline	Pacific Fisheries Environmental Laboratory	Mean values from June, July, August (season when downwelling relaxes)
Lower Trophic Model				
L1	*Chl-a concentration	ug· L^-1^	MODIS	Satellite-derived chla was calculated as an average for April-June, using compiled 8-day satellite chlorophyll-a at a 4 km-resolution from The Hermes GlobColour website [http://hermes.acri.fr/, 26]. This is a standardized merged Chl-a product, combining remote sensing data from SeaWiFS, MERIS, MODIS, VIIRS and OLCI. All calculations were done across the shelf (10m-200m) for the EGOA/WGOA and starting 3 miles offshore
L2	*Chl-a timing of peak bloom	day of year	MODIS	Satellite-derived chla was calculated as an average for April-June, using compiled 8-day satellite chlorophyll-a at a 4 km-resolution from The Hermes GlobColour website [http://hermes.acri.fr/, 26]. This is a standardized merged Chl-a product, combining remote sensing data from SeaWiFS, MERIS, MODIS, VIIRS and OLCI. These data were also used to estimate the timing (day of year) of the maximum spring peak. All calculations were done across the shelf (10m-200m) for the EGOA/WGOA and starting 3 miles offshore
L3	*Mesozooplankton abundance	#· m^-3^	NOAA SE Coastal Monitoring Survey	Calanoid copepod, euphausiid, hyperiid amphipod, and gastropods. Average May-July monthly surveys in Icy Strait, Southeast Alaska; 333 um bongo net (<200m depth)
L4	Copepod community size	ratio	NOAA SE Coastal Monitoring Survey	Number of large calanoid copepods relative to total number of calanoid copepods. Average May-July monthly surveys in Icy Strait, Southeast Alaska; 333 um bongo net (<200m depth); small copepod adults ≤ 2.5mm and large copepods adults > 2.5mm
L5	Large calanoid copepod abundance	#· m^-3^	NOAA SE Coastal Monitoring Survey	Average May-July monthly surveys in Icy Strait, Southeast Alaska; 333 um bongo net (<200m depth); small copepod adults ≤ 2.5mm and large copepods adults > 2.5mm
L6	Small calanoid copepod abundance	#· m^-3^	NOAA SE Coastal Monitoring Survey	Average May-July monthly surveys in Icy Strait, Southeast Alaska; 333 um bongo net (<200m depth); small copepod adults ≤ 2.5mm and large copepods adults > 2.5mm
L7	Euphausiid abundance	#· m^-3^	NOAA SE Coastal Monitoring Survey	Average May-July monthly surveys in Icy Strait, Southeast Alaska; 333 um bongo net (<200m depth)
L8	Hyperiid amphipod abundance	#· m^-3^	NOAA SE Coastal Monitoring Survey	Average May-July monthly surveys in Icy Strait, Southeast Alaska; 333 um bongo net (<200m depth)
L9	Gastropod abundance	#· m^-3^	NOAA SE Coastal Monitoring Survey	Average May-July monthly surveys in Icy Strait, Southeast Alaska; 333 um bongo net (<200m depth)
Mid-Trophic Model				
M1	*CPUE of juvenile pink salmon	CPUE	NOAA SE Coastal Monitoring Survey	CPUE index (log-transformed catch per 20-min trawl set) is the peak monthly average catch rate during the months of June and July, in Icy Strait southeast Alaska
M2	*CPUE of juvenile chum salmon	CPUE	NOAA SE Coastal Monitoring Survey	CPUE index (log-transformed catch per 20-min trawl set) is the peak monthly average catch rate during the months of June and July, in Icy Strait southeast Alaska
M3	*CPUE of juvenile sockeye salmon	CPUE	NOAA SE Coastal Monitoring Survey	CPUE index (log-transformed catch per 20-min trawl set) is the peak monthly average catch rate during the months of June and July, in Icy Strait southeast Alaska
M4	*CPUE of juvenile coho salmon	CPUE	NOAA SE Coastal Monitoring Survey	CPUE index (log-transformed catch per 20-min trawl set) is the peak monthly average catch rate during the months of June and July, in Icy Strait southeast Alaska
M5	*CPUE of juvenile Chinook salmon	CPUE	NOAA SE Coastal Monitoring Survey	CPUE index (log-transformed catch per 20-min trawl set) is the peak monthly average catch rate during the months of June and July, in Icy Strait southeast Alaska
M6	*Mature herring mature biomass in Sitka	tons	Alaska Department of Fish and Game	Estimated mature herring biomass (i.e., pre-fishery biomass) based on integrated statistical catch-at-age models
M7	*Mature herring mature biomass in Craig	tons	Alaska Department of Fish and Game	Estimated mature herring biomass (i.e., pre-fishery biomass) based on integrated statistical catch-at-age models
M8	*Crude birth rate of humpback whales	%	National Park Service	Juvenile humpback encounters in Glacier Bay & Icy Strait, southeast Alaska, relative to total number of whales encountered
Seabird Model				
S1	*Common murre reproductive success	proportion	US Fish and Wildlife Service colony surveys	Proportion of nests with fledged chicks from eggs laid on St. Lazaria Island. Common murre is a diving piscivore.
S2	*Thick-billed murre reproductive success	proportion	US Fish and Wildlife Service colony surveys	Proportion of nests with fledged chicks from eggs laid on St. Lazaria Island. Thick-billed murre is a piscivore.
S3	*Fork-tailed storm petrels reproductive success	proportion	US Fish and Wildlife Service colony surveys	Proportion of nests with fledged chicks from eggs laid on St. Lazaria Island. Fork-tailed storm petrel is a surface planktivore.
S4	*Leach’s storm petrels reproductive success	proportion	US Fish and Wildlife Service colony surveys	Proportion of nests with fledged chicks from eggs laid on St. Lazaria Island. Leach’s storm petrel is a surface planktivore.
S5	*Common murre mean hatch date	day of year	US Fish and Wildlife Service colony surveys	Common murre is a diving piscivore. Day number within year (Jan1 = Day1). St. Lazaria Island.
S6	*Thick-billed murre mean hatch date	day of year	US Fish and Wildlife Service colony surveys	Thick-billed murre is a diving piscivore. Day number within year (Jan1 = Day1). St. Lazaria Island.
S7	*Rhinocerous auklet mean hatch date	day of year	US Fish and Wildlife Service colony surveys	Rhinocerous auklet is a diving zooplanktivore. Day number within year (Jan1 = Day1). St. Lazaria Island.
S8	*Fork-tailed storm petrel mean hatch date	day of year	US Fish and Wildlife Service colony surveys	Fork-tailed storm petrel is a surface zooplanktivore. Day number within year (Jan1 = Day1). St. Lazaria Island.
S9	*Leach’s Storm Petrel mean hatch date	day of year	US Fish and Wildlife Service colony surveys	Leach’s storm petrel is a surface zooplanktivore. Day number within year (Jan1 = Day1). St. Lazaria Island.
S10	*Glaucous-winged gull mean hatch date	day of year	US Fish and Wildlife Service colony surveys	Glaucous-winged gull is a diving piscivore. Day number within year (Jan1 = Day1). St. Lazaria Island.

Some more extensive datasets were subset by species to best represent the diversity of functional groups and to minimize overloading the analyses with similar types of species. For example, a subset of seabird species was selected to represent surface and diving, piscivores and zooplanktivores. Chowiet and St. Lazaria Islands were selected as the representative colonies for the western and eastern GOA, respectively (USFWS dataset, Table 1). The larval groundfish time series were also subset to represent various life histories (EcoFOCI dataset, Table 1). Indicators included both direct measures of ecosystem components (e.g., abundance of forage fish species) and indirect measures (e.g., humpback whale birth rates as indicators of the availability of forage fish as prey). Given the availability of long-term time series for physical oceanography, we analyzed a long-term period (1970–2022) to better identify trends and ecosystem states, and shorter time period (1985–2022) to align with the biological time series. The R script for all analyses is available in a Github repository (https://github.com/ecosystem-state/goa).

### Statistical analyses

We used Dynamic Factor Analysis (DFA) to develop ecosystem-level indicators of GOA climate and biology. DFA models were fit to 92 (55 in western GOA and 37 in eastern GOA) climate and biology indicators described above and in Tables 1 and 2. The DFA model links observed time series ($y_{t}$) to a latent underlying process ($x_{t}$) through an observation equation, $y_{t}=Zx_{t}+v_{t}$ where Z is an estimated loadings matrix that maps latent states to time series, and $v_{t}$ represents white noise $v_{t}\sim MVN(0,R)$. The latent process is modeled as a multivariate random walk, $x_{t}=\;x_{t-1}+w_{t}$ where $w_{t}\sim MVN(0,Q)$ (for identifiability, covariance matrix is fixed as an identity matrix) [27]. We performed estimation in a maximum likelihood framework, using the MARSS package in R [28–30];. For each climate and biology model, we used the AICc model selection criterion to identify (1) data support for the number of latent trends (1 or 2), and (2) the most appropriate variance-covariance matrix (whether the diagonal matrix **R** was a diagonal or not, and whether covariance elements were shared). The maximum number of possible latent trends was limited to two, in order to focus on the study objectives of indicator synthesis and ease in communication.

Non-stationary relationships were identified using moving window cross-correlation analysis. Following the methods of [31], we calculated Pearson correlation coefficients between individual time series and the DFA trends over an 11-year moving window. This window length allowed us to detect non-stationary relationships and capture finer scale trends and variability. Ninety percent confidence intervals were calculated using the Pyper-Peterman correction for autocorrelated data [32]. The relationships were examined for potential changes in sign (change between positive and negative in cases where the confidence intervals do not include a zero value) and/or magnitude (a change greater than 0.5) of the relationship.

Lastly, to identify evidence of state shifts in the ecosystem-level indicators we applied Hidden Markov Models (HMM) to the climate and biology DFA trends. HMM are a class of state-space models that discretize temporal change and are a useful method for detecting underlying states or regimes from noisy time series data [33,34]. HMMs provide estimates of (1) the underlying state or regime at any point in time, (2) the means and variances corresponding to each response in each state, and (3) the transition probabilities between states [34]. We fit two and three-state HMM models to each climate and biology DFA trend and used AICc to identify support for the number of states (maximum three states) [34]. We assumed all response variables were normally distributed. The models were fit using the package hmmTMB [35] in R v. 4.4.1 [[29]]. Code to replicate all of the study analyses are provided in our GitHub repository (https://github.com/ecosystem-state/goa). The models were constricted to maximum three states to fit the frequency of potential changes most suited to fisheries managers.

### Groundtruthing and validation

The DFA and HMM results were groundtruthed by qualitatively comparing model outputs to previously published and observed ecosystem characteristics. The direction of latent trends and associated loadings produced from the DFA during the well-documented marine heatwave years of 2014–2016 and 2019 (hereafter referred to as marine heatwave period) were examined for consistency with published ecological and oceanographic trends [36–38]. HMM results were compared with previously identified potential ecosystem states during the time period of analysis [11,39–41].

## Results

**Fig 2:**
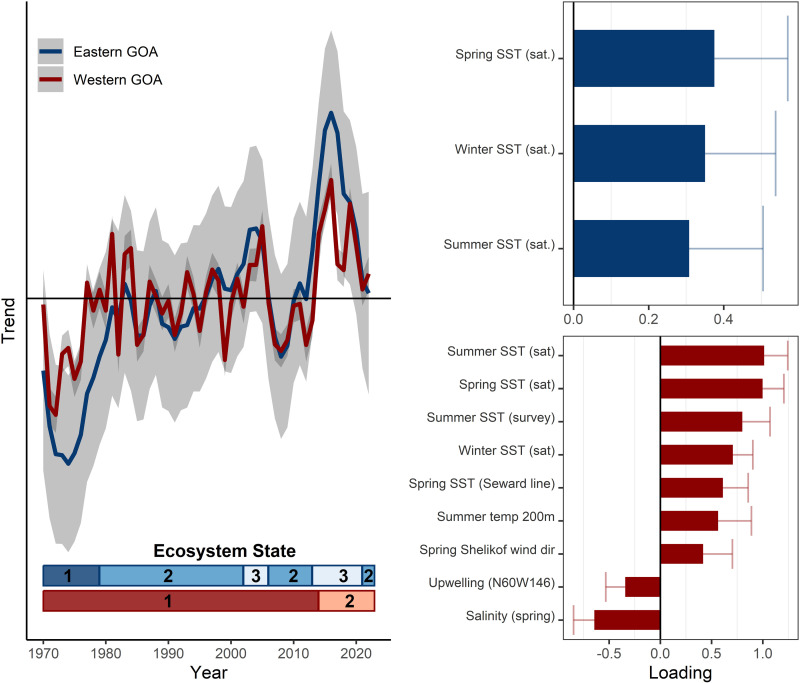
Eastern and western Gulf of Alaska (GOA) model results for the extended climate time series (1970 - 2022). The trends (left) for eastern GOA (blue) and western GOA (red) models are shown with 95% confidence intervals (gray shading). The loadings for variables in the best-fit models (right) for eastern GOA (top) and western GOA (bottom) are shown with 95% confidence intervals (see supplemental for full loading results). Ecosystem states (identified by hidden Markov models) are represented by the horizontal bars for eastern GOA (blue upper bar) and western GOA (red lower bar) at the bottom of the trend plot.

**Fig 3:**
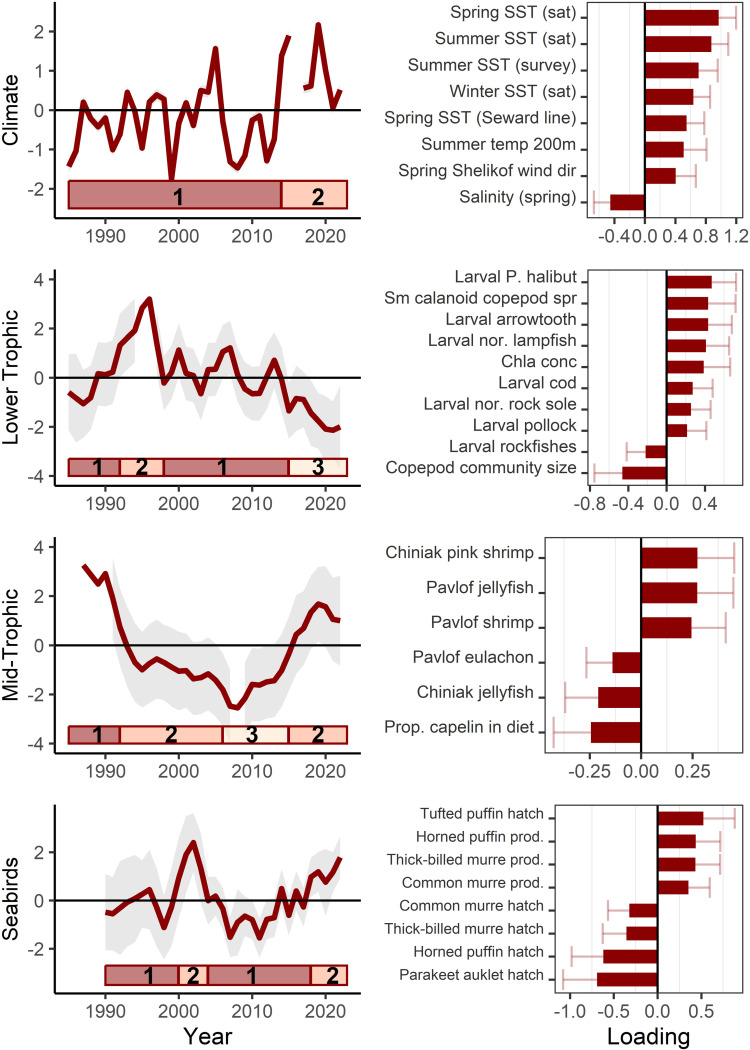
Western Gulf of Alaska model results for climate, lower trophic level, mid-trophic level (1985 - 2022), and seabirds (1990 - 2022). Model trend lines (left) are plotted with 95% confidence intervals shown in gray shading. Ecosystem states (identified by hidden Markov models) are represented by the horizontal bars at the bottom of each trend plot (left). The loadings for variables in the best-fit models (right) are shown with 95% confidence intervals (see supplemental for full loading results).

**Fig 4:**
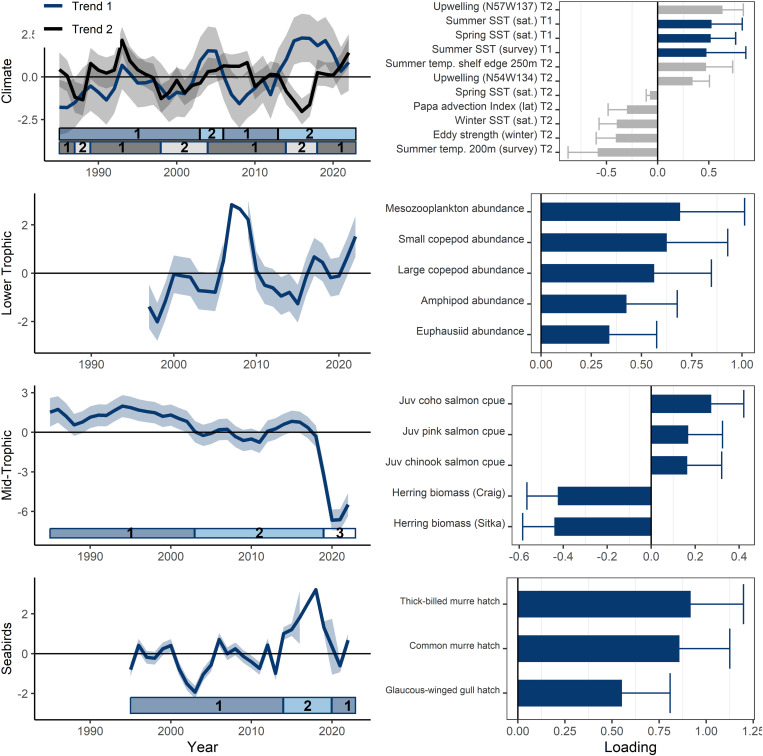
Eastern Gulf of Alaska model results for climate (1985 - 2022), lower trophic level, mid-trophic level (1985 - 2022), and seabirds (1995 - 2022). Model trend lines (left) are plotted with 95% confidence intervals shown in gray shading. The best-fit climate DFA model had two common trends (blue and black). Ecosystem states (identified by hidden Markov models) are represented by the horizontal bars at the bottom of each trend plot (left). The loadings for variables in the best-fit models (right) are shown with 95% confidence intervals (see supplemental for full loading results).

**Fig 5:**
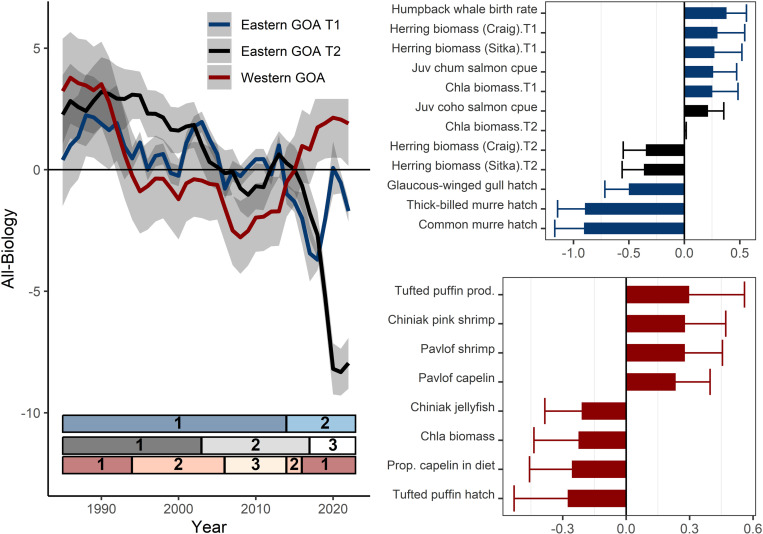
Western (red) and eastern Gulf of Alaska (GOA; blue and black) model results for a subset of all biological indicators (1985 - 2022), including lower trophic level, mid-trophic level, and seabirds. Model trend lines (left) are plotted with 95% confidence intervals shown in gray shading. The best-fit eastern GOA DFA model had two common trends (blue and black). Ecosystem states (identified by hidden Markov models) are represented by the horizontal bars at the bottom of the trend plot (left). The loadings for variables in the best-fit models (eastern GOA: top right, western GOA: bottom right) are shown with 95% upper confidence intervals (see Suppl. Fig 3 for full suite of loading results).

**Fig 6:**
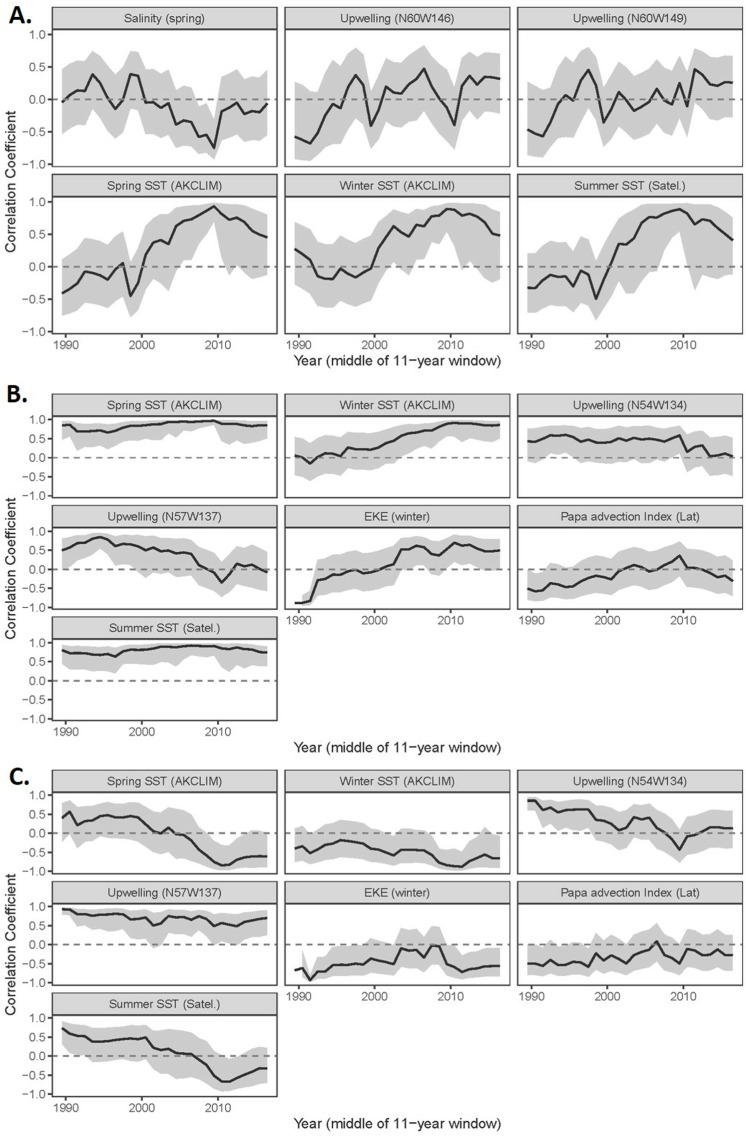
Measures of non-stationarity between individual indicators and DFA trends from the short time series climate models (1985 - 2022) for the (a) western Gulf of Alaska, (b) eastern Gulf of Alaska trend 1, and (c) eastern GOA trend 2.

Results are reported separately for the western and eastern GOA, for each of the climate, lower trophic, mid-trophic, and seabird models. The best-fit DFA models had one trend unless otherwise noted. The DFA results are presented to highlight positive and negative loadings where 95% confidence intervals do not include zero along with the latent trend (Figs 2–5). Full DFA results (including loadings with 95% confidence intervals that do include zero) can be found in S3–S6 Figs. The contribution of each indicator to each ‘trend’ is quantified in direction and magnitude of the ‘loadings. A large loading value identifies indicators that contribute more strongly to the trend. A positive loading would increase/decrease as the trend increases/decreases, while a negative loading has an inverse relationship to the direction of the trend. The direction of latent trends for each model are interpreted for the 2014–2019 marine heatwave period to support validation and interpretation of how well the DFA and HMM models reflected observed environmental and ecological dynamics. The HMM results are presented in terms of number and time period of identified ecosystem states (Figs 2–5). A subset of the results of non-stationarity analyses are presented in Fig 6, with the remainder in the Supplemental Information (S1 and S2 Figs). Transition probabilities for HMM results can be interpreted as the probability of the community moving from one state to another. If the probability of remaining in the state is higher than the probability of transitioning to a new state, then it is more likely to remain in the initial state.

### Western Gulf of Alaska

The latent trend of the long-term period GOA climate model (1970–2022) identified periods of short- and long-term variability (Fig 2). The best-fit model included indicators with positive loadings of ocean temperature at the surface and depth across seasons. Upwelling and salinity loaded negatively. The best-fit HMM model (AICc) identified two ecosystem states: (1) 1970–2013 (probability of staying in state 1 = 0.93), and (2) 2014–2022 (probability of staying in state 2 = 1.00) (Fig 2, S1 Table). The latent trend shows the marine heatwave period achieved the same magnitude as the 1970’s regime shift (~ -2.0). Other warm (positive loading) and cold (negative loading) periods in the time series are of smaller magnitudes.

The climate model of shorter time series (1985–2022) is largely reflective of sea surface temperatures (Fig 3); this result is not surprising as 7 of the 12 time series included represent temperature. The best-fit model included indicators that loaded positive on the latent trend including ocean temperature (winter, spring, summer, surface and at depth) and wind direction in Shelikof Strait. Spring salinity loaded negatively. The latent trend over the 2014–2019 period included warmer, less saline conditions with northeasterly winds in Shelikof Strait (Table 3). The best-fit HMM model (AICc) identified two ecosystem states: 1985–2013 (probability of staying in state 1 = 0.89) and 2014–2022 (probability of staying in state 2 = 0.93) (Fig 3, S1 Table). The model was not sensitive to short-term ecosystem shifts identified in the literature (e.g., 1988, 1998). Non-stationary relationships between individual time series and the latent trend were not evident in terms of changing sign of the relationships (Fig 6).

**Table 3:** Summary of western and eastern GOA model results (DFA trends) during the 2014 - 2016 and 2019 marine heatwave years. Results are summarized from the climate, lower trophic model, mid-trophic level, and seabird models for each region.

Model	Increase	Decrease	Interpretation
**Western GOA**			
Climate	• Sea surface temperature • Shelikof spring wind Direction (west or north)	Spring salinity	Ocean conditions were warmer at the surface, more saline, and spring Shelikof winds were more to the west/north.
Lower Trophic Level	• Copepod community size • Larval rockfish cpue	• Chl-a concentration • Small copepods • Larval pollock, P. cod, arrowtooth flounder, northern lampfish, northern rock sole, P. halibut cpue	Spring primary production was reduced. Larval groundfish cpue was reduced, except for rockfish. Large calanoid copepods increased relative to small copepods.
Mid-Trophic Level	• Chiniak & Pavlof shrimp, Pavlof jellyfish	• Chiniak jellyfish, Pavlof eulachon, proportion of capelin in seabird diets	Some key forage fish (capelin and eulachon) decreased, while shrimp increased.
Seabirds	• Reproductive success (black-legged kittiwakes. common murre, horned puffin, thick-billed murre) and hatch date (later) of tufted puffin	• Hatch date (earlier) for common murre, horned puffin, black-legged kittiwake, parakeet auklet, thick-billed murre	Earlier seabird hatch date (except tufted puffin) and increased reproductive success indicate greater prey availability.
**Eastern GOA**			
Climate	• Sea surface temperature on shelf (summer, spring) • Ocean temperature (~200m on shelf) summer • Eddy kinetic energy • Papa Trajectory Index final latitude • Sea surface temperature on shelf (spring, winter)	• Upwelling index (increased downwelling) • Sea surface temperature on shelf edge, summer	Results imply a strengthened AK current and subtropical gyre and related southerly winds. Ocean temperatures warmed at surface and at depth across all seasons on the shelf (except on the shelf edge). Northward winter surface transport was stronger (Papa Trajectory Index) and eddy strength was stronger (influences along-shelf and cross-shelf transport). Southerly winds and northward surface transport increased Ekman transport that resulted in increased summer downwelling along the coast.
Lower Trophic Level	• Amphipod, ephausiid, mesozooplankton, large copepod, small copepod biomass	• Amphipod, ephausiid, mesozooplankton, large copepod, small copepod biomass	Zooplankton biomass decreased and then increased during this period
Mid-Trophic Level	• Herring biomass	• Juv Chinook, coho, pink, and sockeye salmon cpue	Herring population biomass increased and juvenile salmon cpue decreased during this period.
Seabirds	• Hatch date (later) of common murre, glaucous-winged gull, thick-billed murre (fish-eating surface and diving birds)		Later hatch dates can signify less prey availability in the surrounding waters (i.e., lower abundance of forage fish for these fish-eating seabirds).

The lower trophic level model was largely reflective of chlorophyll-a concentrations and bloom timing and larval groundfish densities (Fig 3). The best-fit model included time series that had positive loadings of chlorophyll-a concentrations, biomass of small calanoid copepods in the spring, larval densities of Pacific halibut, arrowtooth flounder, northern lampfish, Pacific cod, northern rock sole, and pollock. Negatively loading time series included copepod community size (biomass of large calanoid copepods relative to all calanoid copepods) and larval rockfish densities. The latent trend over the 2014–2019 period included increased large calanoid copepods in the spring, increased larval rockfish densities, lower chlorophyll-a concentration, and reduced larval densities of Pacific halibut, arrowtooth flounder, northern lampfish, Pacific cod, northern rock sole, and pollock (Table 3). The best-fit HMM model (AICc) identified three ecosystem states: (1) 1985–1991, 1998–2014 (probability of staying in state 1 = 0.86); (2) 1992–1997 (probability of staying in state 2 = 0.95); and (3) 2015–2022 (probability of staying in state 3 = 0.89) (Fig 3, S1 Table). A non-stationary relationship between the larval northern lampfish time series and the latent trend was evident in terms of changing sign (S1 Fig).

The mid-trophic level model reflects forage species (Fig 3). The best-fit model includes positively loading time series of Chiniak pink shrimp, Pavlof jellyfish, and Pavlof shrimp. Negative loading time series included Chiniak jellyfish, Pavlof eulachon, and the proportion of capelin in the diets of black-legged kittiwake on Middleton Island. The 2014–2019 marine heatwave period reflects a decrease in biomass of cold-affiliated forage species such as capelin and an increase in shrimp (Table 3). The best-fit HMM model (AICc) identified three ecosystem states: (1) 1985–1991 (probability of staying in state 1 = 0.81); (2) 1992–2005, 2015–2022 (probability of staying in state 2 = 0.91); (3) 2006–2014 (probability of staying in state 3 = 1.00) (Fig 3, S1 Table). Non-stationary relationships between individual time series and the latent trend were evident in terms of changing signs (Pavlof capelin, Pavlof eulachon;  S1 Fig)

The seabird model reflected trends in seabird reproductive success (successful fledging of chicks) and hatch date, with little intra-specific differences (Fig 3). Reproductive success of all species loaded positively on the latent trend (black-legged kittiwakes, common murres, fork-tailed storm-petrels, horned puffins, and thick-billed murres). Hatch dates mostly loaded negatively (common murre, horned puffin, black-legged kittiwakes, parakeet auklets, thick-billed murres), with the exception of positive loading tufted puffins. The increasing trend since 2011 reflects progressively earlier seabird hatch dates and increased reproductive success (primarily for those that can lay multiple eggs in successful years). The 2014–2019 marine heatwave period continue the increasing latent trend (Table 3). The best-fit HMM model (AICc) identified two ecosystem states: (1) 1990–1999, 2004–2017 (probability of staying in state 1 = 0.92); (2) 2000–2003, 2018–2022 (probability of staying in state 2 = 0.87) (Fig 3, S1 Table).

The all-biology model reflected trends in a subset of species selected from the lower trophic level, mid-trophic level, and seabird models (Fig 5). The best-fit model includes positive loadings of Tufted puffin reproductive success, capelin and shrimp CPUE in Pavlov Bay. Negative loadings included Tufted puffin hatch dates (earlier), proportion of capelin in seabird diets, and chlorophyll-a concentration. The trend was positive from 1985–1993, was negative through 2015, then positive through the remainder of the time series. The best-fit HMM model (AICc) identified three ecosystem states: (1) 1985–1993 (probability of staying in state 1 = 0.93), (2) 1994–2005, 2014–2015 (probability of staying in state 2 = 0.85), (3) 2016–2022 (probability of staying in state 2 = 0.87) (Fig 5, S1 Table). Non-stationary relationships between individual time series and the latent trends were evident in terms of changing signs (larval sandlance and Pavlof capelin; S1 Fig).

### Eastern GOA

The eastern GOA long-term climate best-fit model (1970–2022) reflected sea surface temperature for winter, spring and summer (positive loadings) (Fig 2). Similar to the western GOA, the latent trend showed the 2014 heating event to be of a similar magnitude to the late 1970’s regime shift. The best-fit HMM model (AICc) identified three ecosystem states: (1) 1970–1978 (probability of staying in state 1 = 0.84); (2) 1979–2001, 2006–2012, 2021–2022 (probability of staying in state 2 = 0.93); (3) 2002–2005, 2013–2020 (probability of staying in state 3 = 0.89) (Fig 2, S1 Table).

The short-term climate best-fit model had two common trends (Fig 4). The first trend reflected spring and summer sea surface temperature (positive loadings). The second trend primarily reflected water transport with positive loadings of coastal upwelling and temperature at depth along the shelf edge, and negative loadings of eddy kinetic energy and Papa Trajectory Index latitude, along with summer temperatures at depth (influenced by upwelling) and winter sea surface temperature. During the 2014–2019 marine heatwave period the first trend increased (aligned with warmer SST) (Table 3). The second trend was negative (2014–2016) and then positive (2017–2019) reflecting fluctuations in water transport dynamics and ocean temperatures at depth over that time period. The best-fit HMM model (AICc) identified two ecosystem states for each trend in the model. Ecosystem states in the first trend included: (1) 1985–2002, 2006–2013 (probability of staying in state 1 = 0.91); and (2) 2003–2005, 2014–2022 (probability of staying in state 2 = 0.92). Ecosystem states in the second trend included were more variable: (1) 1985–1986, 1989–1997, 2004–2013, 2018–2022 (probability of staying in state 1 = 0.86); and (2) 1987–1988, 1998–2003, 2014–2017 (probability of staying in state 2 = 0.72) (Fig 4, S1 Table). The transition years between states did not always align between the two trends. The relationships the latent trend and the eddy kinetic energy (trend 1) and summer SST time series (trend 2), and the latent trend showed evidence of non-stationarity in terms of changing signs (Fig 6).

The lower trophic level model primarily represented zooplankton biomass in the inside waters of Southeast Alaska (Fig 4). The best-fit model include zooplankton groups loading positively to the trend (amphipods, euphausiids, mesozooplankton, large copepods, and small copepods). The best-fit model did not have negative loadings. During the marine heatwave period zooplankton biomass were lower (negative trend: 2014–2016) and then higher (positive trend: 2017–2018), while 2016 and 2019 were close to the mean (Table 3). The ecosystem state analysis (HMM) did not converge due to the shorter time series.

The mid-trophic level best-fit model also had one trend (Fig 4). This was an inverse relationship between herring biomass (negative loading) and juvenile salmon (coho, pink, Chinook: positive loading). The trend was positive from 2014–2017 but started a precipitous decline in 2017, reflecting the low survival of juvenile salmon and the strong year classes of herring during that warm period (Table 3). The best-fit HMM model (AICc) identified three ecosystem states (1) 1985–2002 (probability of staying in state 1 = 0.94); (2) 2003–2018 (probability of staying in state 2 = 1.00); and (3) 2019–2022 with a relatively large change in the trend value (probability of staying in state 3 = 1.00) (Fig 4, S1 Table). There was no evidence of non-stationarity in this model (S2 Fig).

The seabird model reflected hatch dates of fish-eating surface and diving birds (common murres, glaucous-winged gulls, and thick-billed murres, but not rhinoceros auklets) (Fig 4). Hatch dates of zooplankton-eating seabirds (fork-tailed and Leach’s storm-petrels) were not included in the best-fit model. The eastern GOA latent trend did not show the same long-term increasing trend (earlier hatch dates) as the western GOA. The trend decreased in the 2014–2019 warm period, signifying early hatch dates for fish-eating seabirds (Table 3). The best-fit HMM model (AICc) identified two ecosystem states: 1995–2013, 2020–2022 (probability of staying in state 1 = 0.82); and (2) 2014–2019 (probability of staying in state 2 = 0.95) (Fig 4, S1 Table).

The all-biology best-fit model had two common trends (Fig 5). The model reflected trends in a subset of species selected from the lower trophic level, mid-trophic level, and seabird models (Fig 5). The best-fit model included positive loadings on the first trend of humpback whale crude birth rate, herring biomass, juvenile chum salmon CPUE, chlorophyll-a concentration. Negative loadings on the first trend include hatch dates (earlier dates) of glaucous-winged gulls, thick-billed murre, and common murres. The best-fit model included negative loadings on the second trend of herring biomass and juvenile coho salmon CPUE. The best-fit HMM model (AICc) identified two ecosystem states for the first trend: 1985–2013 (probability of staying in state 1 = 0.97) and 2014–2022 (probability of staying in state 2 = 1.00). The best-fit HMM model (AICc) identified three ecosystem states for the second trend: 1985–2002 (probability of staying in state 1 = 0.94), 2003–2016 (probability of staying in state 2 = 0.94), and 2017–2022 (probability of staying in state 3 = 1.00) (Fig 5, S1 Table). There was no evidence of non-stationary relationships between individual time series and either of the latent trends (S2 Fig).

## Discussion

The use of statistical tools to integrate marine ecosystem time series can aid in identifying latent ecosystem trends, depict changes in ecosystem states, and provide streamlined communication in support of fisheries management. In the Gulf of Alaska, multiple sets of ecosystem indicators covaried within the same climate and functional group model (e.g., zooplankton, seabirds), indicating the potential for reducing the core number of indicators required to communicate the common ecosystem trend. Ecosystem states, identified in the western and eastern GOA, highlight the ability of the current suite of time series to reflect multi-year changes throughout the ecosystem. These tools can be operationalized in Gulf of Alaska fisheries management to improve synthesis and communication of ecosystem information, and to implement the concept of ecosystem states via multi-year perspectives on ecosystem conditions.

The identification of latent trends across suites of ecosystem indicators in the GOA provides a basis upon which to synthesize and prioritize the communication of ecosystem information in support of groundfish fisheries management. Currently a large number of ecosystem indicators are included in the annual Gulf of Alaska Ecosystem Status Report, produced to provide ecosystem-wide information external to stock assessment models to the harvest specification process [1,15]. It is important to collect indicator information at finer temporal and spatial scales to be able to understand changes in the latent trends, to have the opportunity to highlight specific areas (e.g., spawning areas), seasons (e.g., spring for larval groundfish), and intra-group changes in variability (e.g., seabird reproductive success across species and colonies), and because the ecological importance of indicators can change over time [21,42–45]. However, the application of DFA provides a method to curate this information to aid in its communication. The DFA identified suites of time series in the western and eastern GOA that covaried across spatial scales (e.g., area-specific ocean temperature surveys) and species-specific scales (e.g., biomass of zooplankton and reproductive success and phenology of seabirds) that better matches the GOA groundfish ecosystem and management scale of interest. These covarying indicators would be strong contenders to use the latent trends instead of individual trends to synthesize and communicate ecosystem status to managers.

The latent trends in most of the western and eastern GOA models reflected observed trends and shifts of individual indicators during key environmental periods, including the marine heatwave. The 2014 marine heatwave aligned with a change in ecosystem state across all the western and eastern GOA models (except the eastern GOA mid-trophic level model), matching previously documented ecosystem-wide responses (e.g. [37,38],). The western GOA models identified warmer, more saline waters, later and reduced spring bloom, and reduced larval groundfish (increased larval rockfish being the exception), consistent with observations during the 2014–2016 marine heatwave years [46]. The western GOA mid-trophic level model identified a decline in forage species, reflecting observed declines in capelin [47]. The trend does not reflect the full forage species narrative during this period as it does not represent all the relevant species (e.g., herring, sandlance) and their inverse relationships with each other [48]. This western GOA mid-trophic model is a cautionary note to understand the representativeness of indicators in interpretation of these model results [16,49,50]. The eastern GOA models match some known conceptual models including the oceanography characterized by ocean temperature (first model trend) and by ocean transport (second model trend: Papa Trajectory Index, eddy strength, downwelling strength). The eastern GOA mid-trophic level and seabird model trends follow observed trajectories of individual populations but present opportunities to further explore non-intuitive results (e.g., the inverse relationships of herring and juvenile salmon).

**Fig 7:**
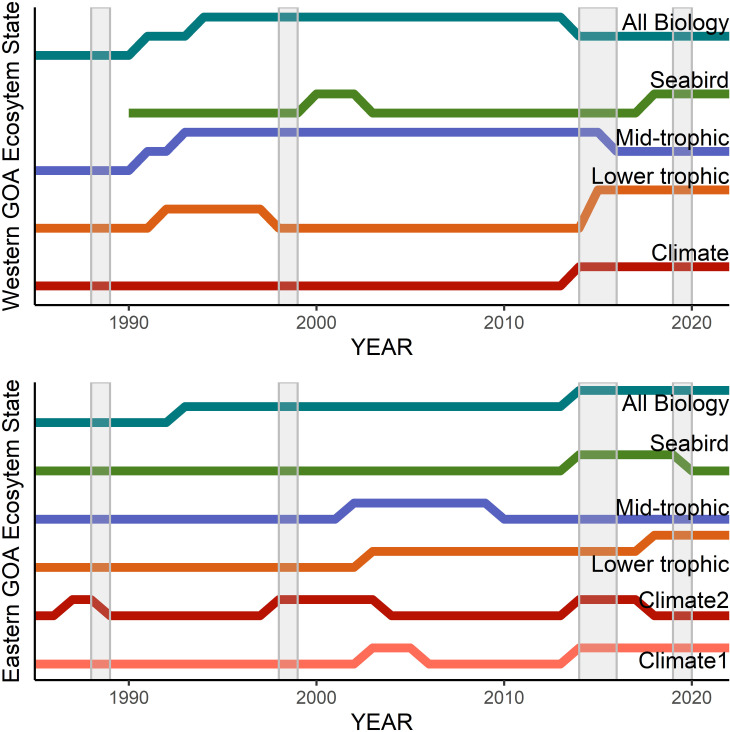
Ecosystem States across climate, lower trophic level, mid-trophic level, and seabird and all biology models from 1985 - 2022 in the western (top) and eastern (bottom) Gulf of Alaska. Previously documented ecosystem changes are shown as gray bars (see discussion for citations). Ecosystem states all have a probability > 0.8 of remaining within that state.

The HMM analysis identified two to three ecosystem states throughout the western and eastern GOA models (Fig 7). The ability of the analyses to detect ecosystem states is validated by comparing the HMM results with previously observed long-term and short-term ecosystem shifts. The western and eastern GOA long-term climate model (1970–2022) identified the 2014–2019 marine heatwave period as being equal in magnitude of deviation from the mean as the 1976/1977 period, a well-documented regime shift in the region [11,39,40]. The western GOA models identified 1988/1989 ecosystem state changes in the all-biology and mid-trophic level models, and a delayed change in the lower trophic level models, but no change in the climate models. This pattern of signal in the biology, but not climate data, aligns with the conclusions of other analyses of the period [40,41]. The weaker climate signal for the 1989 regime shift may have been due to key environmental data missing from the analyses or the event being driven by a short-lived or less extreme physical change that had persistent implications throughout the ecosystem. In the eastern GOA, this shift was only observed in the second trend of the climate model (indicators related to water transport). The 1998/1999 [51,52] ecosystem state change was identified in the western GOA lower trophic level and seabirds, but not the mid-trophic level and all-biology model. The 2014 ecosystem state shift [36,53] was identified in all our western and eastern GOA models, with a delayed eastern GOA mid-trophic level change, agreeing with the broad ecosystem dynamics documented from that time period [12,37,47,54]. Our models show the 2014 ecosystem state persisting through 2022. This finding adds to the ongoing discussion of persistence in changes of ecosystem state, including the finding of no persistent change [55] and the contrary finding of persistent change through 2019 [ [38]]. Interestingly, some documented GOA ecosystem state changes were not detected in the eastern GOA, perhaps reflecting the reliance on western GOA ecological time series to define previous states (e.g., Alaska Department of Fish and Game small-mesh trawl [11],). These ecosystem state results show the sensitivity of the models to detect previously observed short-term ecosystem changes, validating this tool for future applications. The results also highlight the importance of monitoring indicators throughout the food web (climate, and varying ecological subgroups) and across spatial gradients (e.g., eastern and western GOA) to detect the presence of ecosystem state shifts and the magnitude of response through the system [11].

Operationalization of the DFA and HMM tools in support of Gulf of Alaska fisheries management will aid in the interpretation and early warning of ecosystem status and changes that are relevant to groundfish productivity. These tools and concepts could be added to the existing framework for communicating ecosystem information to the regional fisheries management council during their annual groundfish harvest specification process, including GOA Ecosystem Status Reports [15], risk tables [14], and Ecosystem and Socioeconomic Profiles [13]. The existing annual process of updating the GOA Ecosystem Status Report involves updating all ecosystem indicators (including those used in this study), primarily with data collected in the same calendar year. This framework could be rapidly expanded to include annual updates of the DFA and HMMs to identify static or changed relationships within the ecosystem (loadings and non-stationarity) and potential of continued or changed ecosystem states. These tools would provide qualitative insight into the potential changes in fish productivity and survival in the coming year, similar to efforts in the California Current fisheries management system [9]. Any changes in relationships between individual time series (non-stationary relationships) would inform a more focused examination of those data to identify unexpected changes in the ecosystem. Communication of this ecosystem information to the regional fisheries management council would include synthesizing the direction and magnitude of latent trends to explain conditions of groundfish environment, predators, prey, and competitors, relative to past years. An ecosystem that is moving to a new state might warrant more precaution (due to higher uncertainty) than one returning to a previously observed state. The ecosystem state change could be interpreted based on its detection across multiple ecosystem models (e.g., lower trophic level, mid-trophic level, seabirds), and whether it is detected in the western and/or eastern GOA, to inform the magnitude of change and potential risk or benefit to groundfish. Additionally, if a change is detected, managers can expect greater uncertainty in the coming year and incorporate more precautionary decisions where appropriate. Examination of results from the 2014 marine heatwave period shows these tools could have provided are more synthesized, early warning message of broad ecosystem changes throughout the ecosystem, in the context of previous changes in ecosystem state.

The DFA and HMM are useful tools to support ecosystem-based management. A key challenge to the management application of these tools is the development of a response to a known ecosystem state change with unknown knowledge of its future persistence. Another challenge is the interpretation of the ecosystem state concept in combination with underlying gradual (non-abrupt) changes in the ecosystem that would not be detected by this tool [56,57]. Length of time series and data availability limit the breadth of ecosystem components that can be included and synthesized, and non-stationarity and changes in environmental-biological relationships can change the relative importance of individual indicators. However, in the context of using best available science to inform fisheries management, there is value in adding these tools to the ecosystem-based fisheries management toolbox to aid in interpretation of ecosystem information and early warning of transitions [58]. Greater clarity of ecosystem trends and states will aid communication of ecosystem status and provide context for the level of risk associated with harvest decisions, as well as early warnings for potential ecosystem changes that might affect future fisheries. DFA analyses help focus research on key mechanistic relationships to advance our understanding and ability to predict outcomes. Future research to improve the application of these tools include connecting GOA DFA models to climate forecasts, connecting DFA models and ecosystem states to trends in groundfish recruitment, and exploration of thresholds and tipping points [9,24,59,60].

## Supporting information

S1 Table Summary of HMM results for eastern and western GOA models to determine the number of ecosystem states (AICc; bolded) and the probability of remaining in the same state. (DOCX)

S1 Fig Measures of non-stationarity between individual indicators and DFA trends in the western GOA, including the (A) lower-trophic level model, (B) mid-trophic level model, and (C) all biology model. (DOCX)

S2 Fig Measures of non-stationarity between individual indicators and DFA trends in the eastern GOA including the (A) mid-trophic level model, (B) all biology model (Trend 1), and (C) all biology model (Trend 2). (DOCX)

S3 Fig All loadings from western GOA DFA analyses of the (A) climate, (B) lower trophic, (C) mid-trophic, and (D) seabird models. (DOCX)

S4 Fig All loadings from western GOA DFA analyses of the all biology model. (DOCX)

S5 Fig All loadings from eastern GOA DFA analyses of the (A) climate (trend 1, T1, in blue and trend 2, T2, in red), (B) lower trophic, (C) mid-trophic, and (D) seabird models. (DOCX)

S6 Fig All loadings from eastern GOA DFA analyses of the all biology model’s trend 1 (T1, black) and trend 2 (T2, red). (DOCX)
