# Supplementary material for: Identifying common trends and ecosystem states to inform Gulf of Alaska ecosystem-based fisheries management
Source: PLoS One. 2025 Jun 6;20(6):e0324154. doi: 10.1371/journal.pone.0324154 (PMC12143537; doi:10.1371/journal.pone.0324154)
Supplement: S1 Table — (DOCX) [file pone.0324154.s001.docx]

**Identifying Common Trends and Ecosystem States to Inform Gulf of Alaska Ecosystem-Based Fisheries Management**

Bridget E. Ferriss^1^, Mary E. Hunsicker^2^, Eric J. Ward^3^, Michael A. Litzow^4^, Lauren Rogers^5^, Matt Callahan^6^, Wei Cheng^7^, Seth L. Danielson^8^, Brie Drummond^9^, Emily Fergusson^10^, Christine Gabriele^11^, Kyle Hebert^12^, Russell R. Hopcroft^13^, Jens Nielsen^5,14^, Kally Spalinger^15^, William T. Stockhausen^1^, Wesley W. Strasburger^10^, Shannon Whelan^16^

^1^Resource Ecology and Fisheries Management Division, Alaska Fisheries Science Center, National Marine Fisheries Service, National Oceanic and Atmospheric Administration, Seattle, WA, USA

^2^Fish Ecology Division, Northwest Fisheries Science Center, National Marine Fisheries Service, National Oceanic and Atmospheric Administration, Newport, OR, USA

^3^Conservation Biology Division, Northwest Fisheries Science Center, National Marine Fisheries Service, National Oceanic and Atmospheric Administration, Seattle, WA, USA

^4^Shellfish Assessment Program, Resource Assessment and Conservation Engineering Division, Alaska Fisheries Science Center, National Marine Fisheries Service, National Oceanic and Atmospheric Administration, Kodiak, AK, USA

^5^Resource Assessment and Conservation Engineering Division, Alaska Fisheries Science Center, National Marine Fisheries Service, National Oceanic and Atmospheric Administration, Seattle, WA, USA

^6^Pacific States Marine Fisheries Commission, Alaska Fish Information Network, Juneau, AK, USA

^7^Pacific Marine Environmental Laboratory, National Oceanic and Atmospheric Administration, Seattle, WA, USA

^8^College of Fisheries and Ocean Sciences, University of Alaska Fairbanks, Fairbanks, AK 99775-7220, USA

^9^U.S. Fish and Wildlife Service, Alaska Maritime National Wildlife Refuge, Homer, AK, USA

^10^Auke Bay Laboratories Division, Alaska Fisheries Science Center, National Marine Fisheries Service, National Oceanic and Atmospheric Administration, Juneau, AK, USA

^11^Glacier Bay National Park and Preserve, Gustavus, AK, USA

^12^Alaska Department of Fish and Game, Commercial Fisheries Division, Juneau, AK, USA

^13^College of Fisheries and Ocean Sciences, University of Alaska Fairbanks, Fairbanks, AK 99775-7220, USA

^14^Cooperative Institute for Climate, Ocean, and Ecosystem Studies, University of Washington, Seattle, WA, USA

^15^Alaska Department of Fish and Game, Commercial Fisheries Division, Kodiak, AK, USA

^16^Institute for Seabird Research and Conservation, Anchorage, AK, USA

Suppl. Table 1. Summary of HMM results for eastern and western GOA models to determine the number of ecosystem states (AICc; bolded) and the probability of remaining in the same state.

| Model | Trend # | States # | AICc | Probability of remaining within state | | |
| --- | --- | --- | --- | --- | --- | --- |
|  |  |  |  | State 1 | State 2 | State 3 |
| Eastern GOA | |  |  |  |  |  |
| All biology | 1 | 2 | **121.0558** | 0.9655 | 1.0000 |  |
|  |  | 3 | 119.3759 |  |  |  |
|  | 2 | 2 | 164.2289 |  |  |  |
|  |  | 3 | **122.3077** | 0.9442 | 0.9377 | 1.0000 |
| Climate (long) | 1 | 2 | 175.7649 |  |  |  |
|  |  | 3 | **150.9815** | 0.8443 | 0.9320 | 0.8945 |
| Climate (short) | 1 | 2 | **104.7137** | 0.9047 | 0.9166 |  |
|  |  | 3 | 106.5261 |  |  |  |
|  | 2 | 2 | **99.6681** | 0.8623 | 0.7152 |  |
|  |  | 3 | 109.1107 |  |  |  |
| Lower trophic | 1 | 2 | did not converge | |  |  |
|  |  | 3 | did not converge | |  |  |
| Mid-trophic | 1 | 2 | 115.8626 |  |  |  |
|  |  | 3 | **92.5168** | 0.9430 | 1.0000 | 1.0000 |
| Seabirds | 1 | 2 | **86.6238** | 0.8189 | 0.9501 |  |
|  |  | 3 | 95.6803 |  |  |  |
| Western GOA | |  |  |  |  |  |
| All biology | 1 | 2 | 127.0923 |  |  |  |
|  |  | 3 | **113.9239** | 0.9339 | 0.8542 | 0.8730 |
| Climate (long) | 1 | 2 | **149.3367** | 0.9773 | 1.0000 |  |
|  |  | 3 | 150.3032 |  |  |  |
| Climate (short) | 1 | 2 | **109.5083** | 0.8945 | 0.9322 |  |
|  |  | 3 | 119.8972 |  |  |  |
| Lower trophic | 1 | 2 | 123.7922 |  |  |  |
|  |  | 3 | **117.5581** | 0.8570 | 0.9515 | 0.8933 |
| Mid-trophic | 1 | 2 | 138.482 |  |  |  |
|  |  | 3 | **120.6338** | 0.8108 | 0.9143 | 1.0000 |
| Seabirds | 1 | 2 | **86.40937** | 0.9152 | 0.8690 |  |
|  |  | 3 | 100.4094 |  |  |  |
