# Supplementary material for: Identifying common trends and ecosystem states to inform Gulf of Alaska ecosystem-based fisheries management
Source: PLoS One. 2025 Jun 6;20(6):e0324154. doi: 10.1371/journal.pone.0324154 (PMC12143537; doi:10.1371/journal.pone.0324154)
Supplement: S2 Fig — (DOCX) [file pone.0324154.s003.docx]

**Identifying Common Trends and Ecosystem States to Inform Gulf of Alaska Ecosystem-Based Fisheries Management**

Bridget E. Ferriss^1^, Mary E. Hunsicker^2^, Eric J. Ward^3^, Michael A. Litzow^4^, Lauren Rogers^5^, Matt Callahan^6^, Wei Cheng^7^, Seth L. Danielson^8^, Brie Drummond^9^, Emily Fergusson^10^, Christine Gabriele^11^, Kyle Hebert^12^, Russell R. Hopcroft^13^, Jens Nielsen^5,14^, Kally Spalinger^15^, William T. Stockhausen^1^, Wesley W. Strasburger^10^, Shannon Whelan^16^

^1^Resource Ecology and Fisheries Management Division, Alaska Fisheries Science Center, National Marine Fisheries Service, National Oceanic and Atmospheric Administration, Seattle, WA, USA

^2^Fish Ecology Division, Northwest Fisheries Science Center, National Marine Fisheries Service, National Oceanic and Atmospheric Administration, Newport, OR, USA

^3^Conservation Biology Division, Northwest Fisheries Science Center, National Marine Fisheries Service, National Oceanic and Atmospheric Administration, Seattle, WA, USA

^4^Shellfish Assessment Program, Resource Assessment and Conservation Engineering Division, Alaska Fisheries Science Center, National Marine Fisheries Service, National Oceanic and Atmospheric Administration, Kodiak, AK, USA

^5^Resource Assessment and Conservation Engineering Division, Alaska Fisheries Science Center, National Marine Fisheries Service, National Oceanic and Atmospheric Administration, Seattle, WA, USA

^6^Pacific States Marine Fisheries Commission, Alaska Fish Information Network, Juneau, AK, USA

^7^Pacific Marine Environmental Laboratory, National Oceanic and Atmospheric Administration, Seattle, WA, USA

^8^College of Fisheries and Ocean Sciences, University of Alaska Fairbanks, Fairbanks, AK 99775-7220, USA

^9^U.S. Fish and Wildlife Service, Alaska Maritime National Wildlife Refuge, Homer, AK, USA

^10^Auke Bay Laboratories Division, Alaska Fisheries Science Center, National Marine Fisheries Service, National Oceanic and Atmospheric Administration, Juneau, AK, USA

^11^Glacier Bay National Park and Preserve, Gustavus, AK, USA

^12^Alaska Department of Fish and Game, Commercial Fisheries Division, Juneau, AK, USA

^13^College of Fisheries and Ocean Sciences, University of Alaska Fairbanks, Fairbanks, AK 99775-7220, USA

^14^Cooperative Institute for Climate, Ocean, and Ecosystem Studies, University of Washington, Seattle, WA, USA

^15^Alaska Department of Fish and Game, Commercial Fisheries Division, Kodiak, AK, USA

^16^Institute for Seabird Research and Conservation, Anchorage, AK, USA

A.


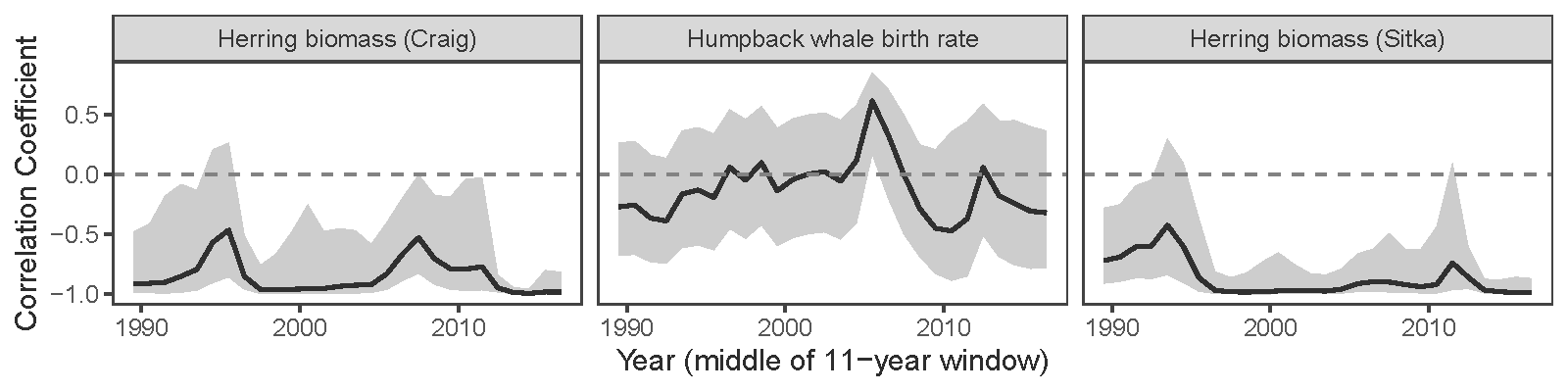


B.
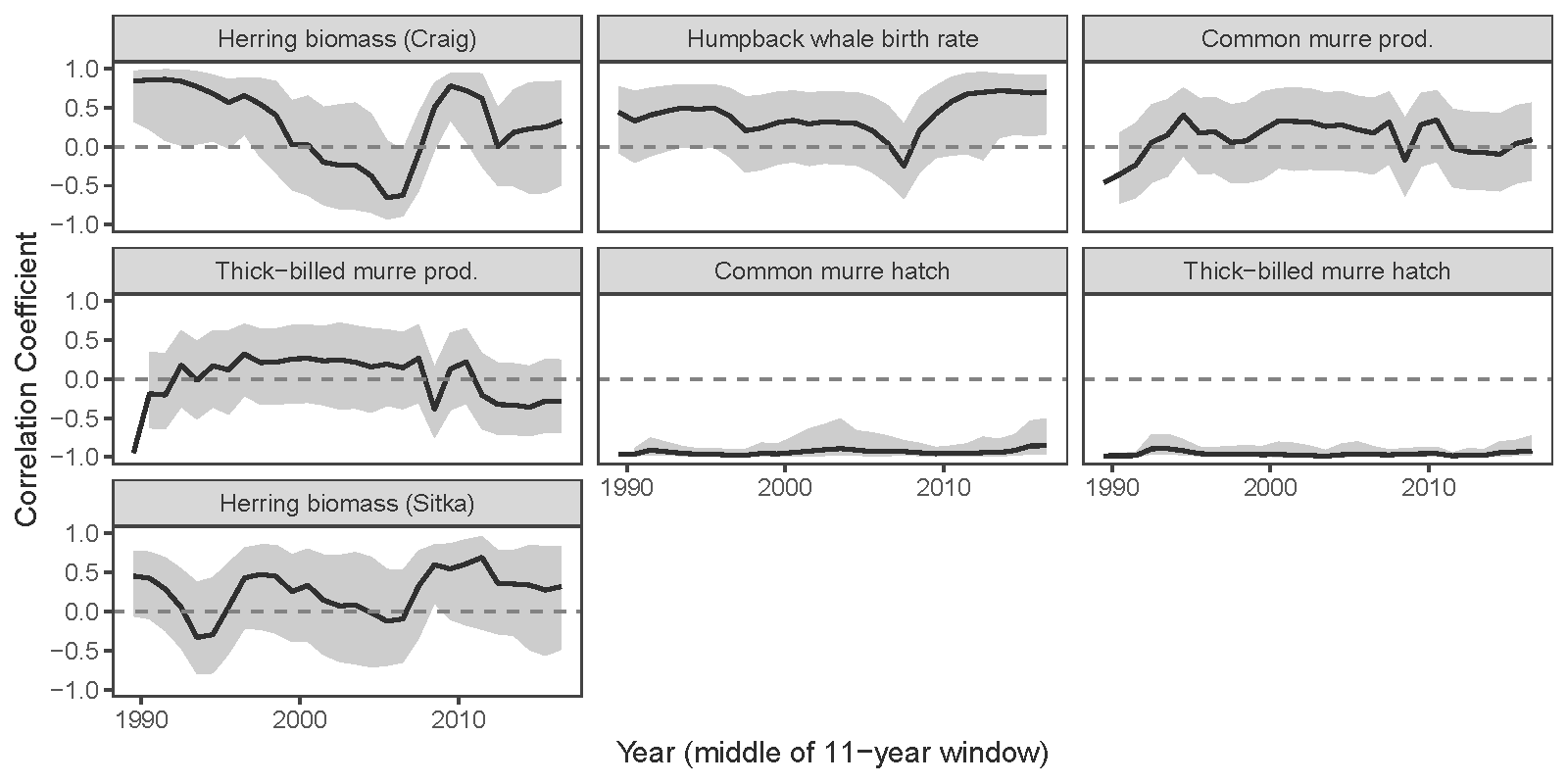


C.
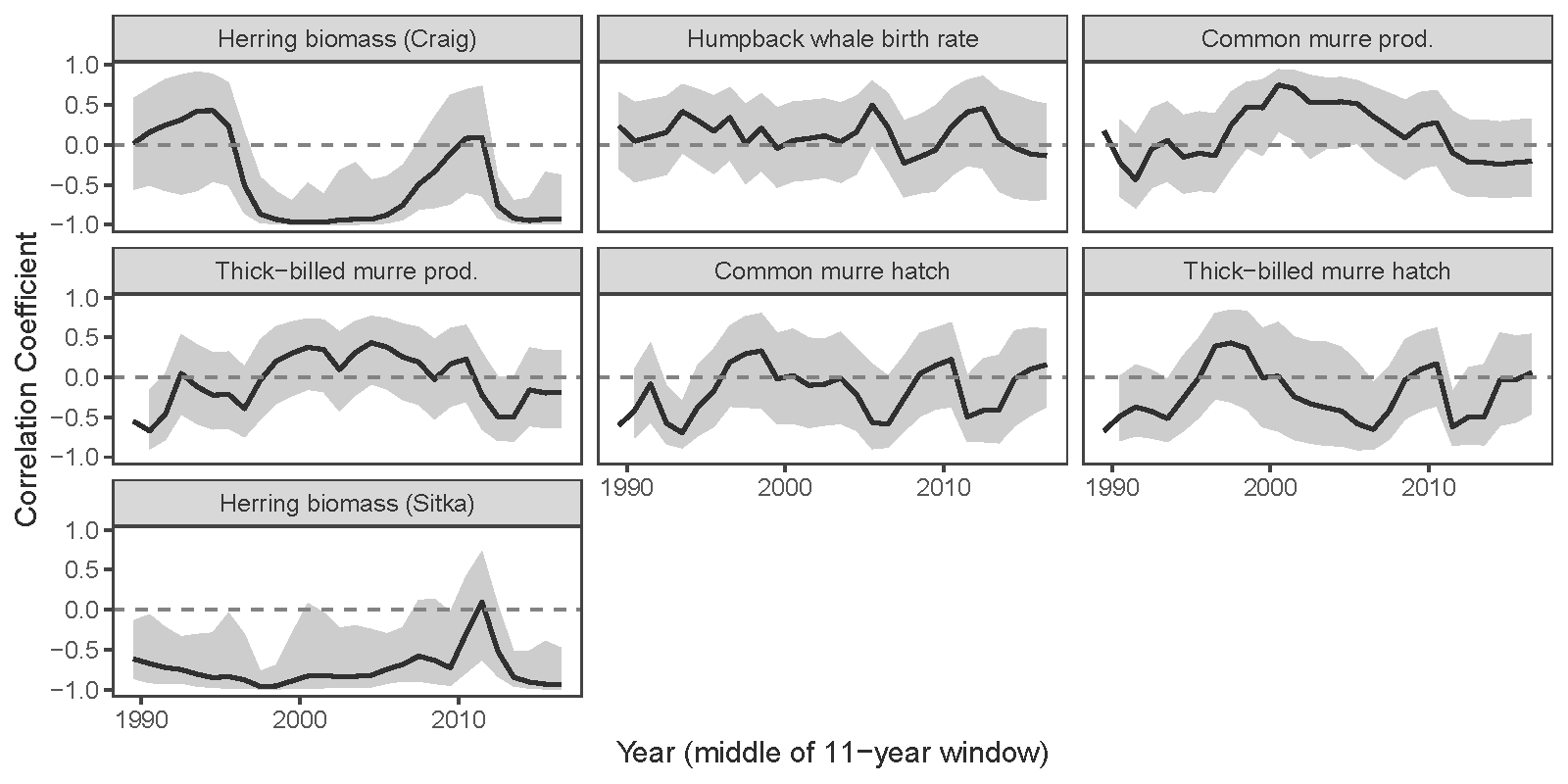


Suppl. Figure 2. Measures of non-stationarity between individual indicators and DFA trends in the eastern GOA including the (A) mid-trophic level model, (B) all biology model (Trend 1), and (C) all biology model (Trend 2).
