# Supplementary material for: Identifying common trends and ecosystem states to inform Gulf of Alaska ecosystem-based fisheries management
Source: PLoS One. 2025 Jun 6;20(6):e0324154. doi: 10.1371/journal.pone.0324154 (PMC12143537; doi:10.1371/journal.pone.0324154)
Supplement: S5 Fig — (DOCX) [file pone.0324154.s006.docx]

**Identifying Common Trends and Ecosystem States to Inform Gulf of Alaska Ecosystem-Based Fisheries Management**

Bridget E. Ferriss^1^, Mary E. Hunsicker^2^, Eric J. Ward^3^, Michael A. Litzow^4^, Lauren Rogers^5^, Matt Callahan^6^, Wei Cheng^7^, Seth L. Danielson^8^, Brie Drummond^9^, Emily Fergusson^10^, Christine Gabriele^11^, Kyle Hebert^12^, Russell R. Hopcroft^13^, Jens Nielsen^5,14^, Kally Spalinger^15^, William T. Stockhausen^1^, Wesley W. Strasburger^10^, Shannon Whelan^16^

^1^Resource Ecology and Fisheries Management Division, Alaska Fisheries Science Center, National Marine Fisheries Service, National Oceanic and Atmospheric Administration, Seattle, WA, USA

^2^Fish Ecology Division, Northwest Fisheries Science Center, National Marine Fisheries Service, National Oceanic and Atmospheric Administration, Newport, OR, USA

^3^Conservation Biology Division, Northwest Fisheries Science Center, National Marine Fisheries Service, National Oceanic and Atmospheric Administration, Seattle, WA, USA

^4^Shellfish Assessment Program, Resource Assessment and Conservation Engineering Division, Alaska Fisheries Science Center, National Marine Fisheries Service, National Oceanic and Atmospheric Administration, Kodiak, AK, USA

^5^Resource Assessment and Conservation Engineering Division, Alaska Fisheries Science Center, National Marine Fisheries Service, National Oceanic and Atmospheric Administration, Seattle, WA, USA

^6^Pacific States Marine Fisheries Commission, Alaska Fish Information Network, Juneau, AK, USA

^7^Pacific Marine Environmental Laboratory, National Oceanic and Atmospheric Administration, Seattle, WA, USA

^8^College of Fisheries and Ocean Sciences, University of Alaska Fairbanks, Fairbanks, AK 99775-7220, USA

^9^U.S. Fish and Wildlife Service, Alaska Maritime National Wildlife Refuge, Homer, AK, USA

^10^Auke Bay Laboratories Division, Alaska Fisheries Science Center, National Marine Fisheries Service, National Oceanic and Atmospheric Administration, Juneau, AK, USA

^11^Glacier Bay National Park and Preserve, Gustavus, AK, USA

^12^Alaska Department of Fish and Game, Commercial Fisheries Division, Juneau, AK, USA

^13^College of Fisheries and Ocean Sciences, University of Alaska Fairbanks, Fairbanks, AK 99775-7220, USA

^14^Cooperative Institute for Climate, Ocean, and Ecosystem Studies, University of Washington, Seattle, WA, USA

^15^Alaska Department of Fish and Game, Commercial Fisheries Division, Kodiak, AK, USA

^16^Institute for Seabird Research and Conservation, Anchorage, AK, USA


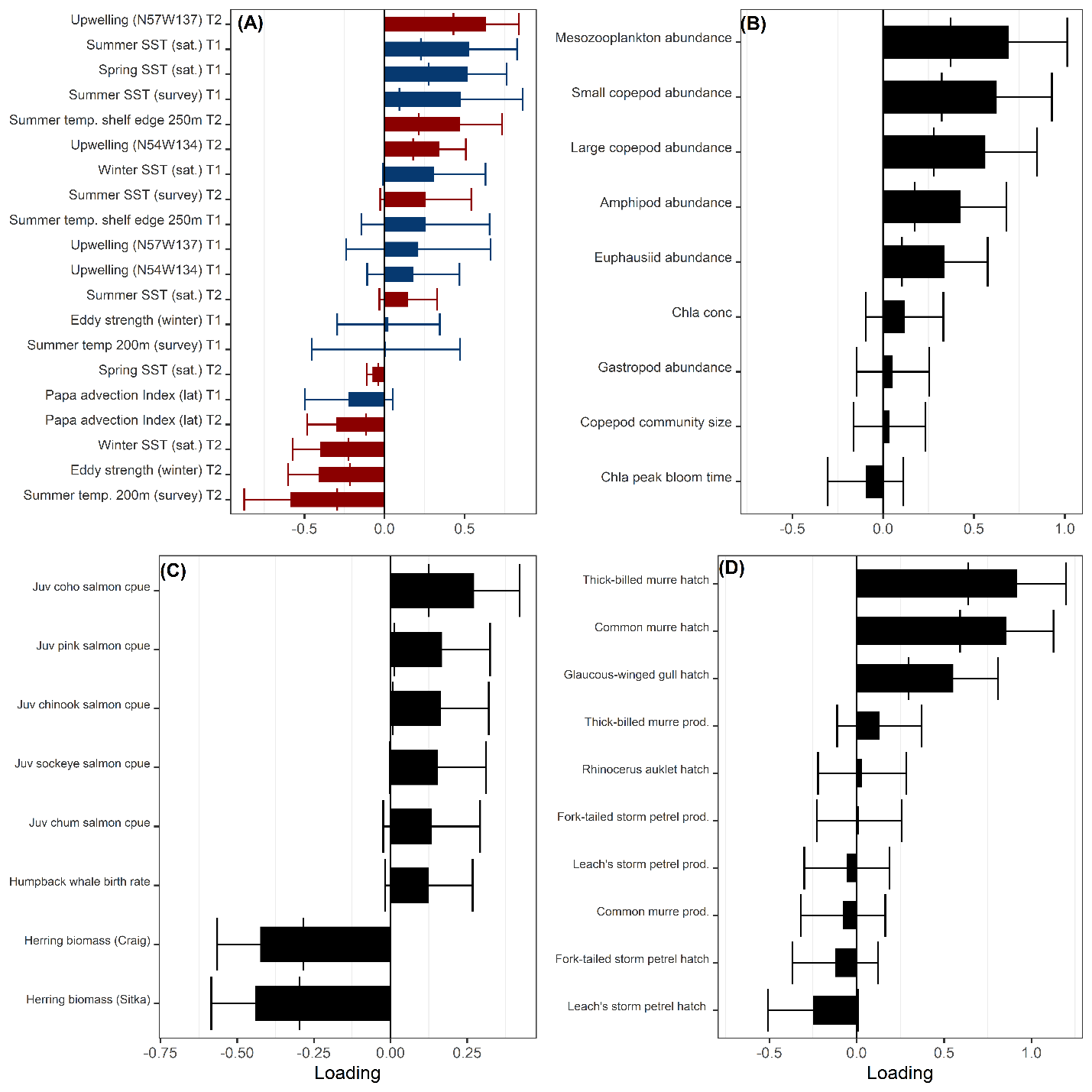


Suppl. Figure 5. All loadings from eastern GOA DFA analyses of the (A) climate (trend 1, T1, in blue and trend 2, T2, in red), (B) lower trophic, (C) mid-trophic, and (D) seabird models.
